# Targeting inhibition of extracellular signal-regulated kinase kinase pathway with AZD6244 (ARRY-142886) suppresses growth and angiogenesis of gastric cancer

**DOI:** 10.1038/srep16382

**Published:** 2015-11-16

**Authors:** Jin-Hang Gao, Chun-Hui Wang, Huan Tong, Shi-Lei Wen, Zhi-Yin Huang, Cheng-Wei Tang

**Affiliations:** 1 Division of Peptides Related with Human Diseases, State Key Laboratory of Biotherapy, West China Hospital, Sichuan University, Chengdu, China; 2 Department of Gastroenterology, West China Hospital, Sichuan University, Chengdu, China; 3 Department of Human Anatomy, Academy of Preclinical and Forensic Medicine, Sichuan University, Chengdu, China

## Abstract

AZD6244 (ARRY-142886), a highly selective MAPK-ERK kinase inhibitor, has shown excellent clinical efficacy in many tumors. However, the anti-tumor and anti-angiogenesis efficacy of AZD6244 on gastric cancer has not been well characterized. In this study, high p-ERK expression was associated with advanced TNM stage, increased lymphovascular invasion and poor survival. For absence of NRAS, KRAS and BRAF mutation, SGC7901 and BGC823 gastric cancer cells were relative resistance to AZD6244 *in vitro*. And such resistance was not attributed to the insufficient inhibition of ERK phosphorylation. However, tumor growth was significantly suppressed in SGC7901 xenografts by blockage of angiogenesis. This result was further supported by suppression of tube formation and migration in HUVEC cells after treatment with AZD6244. Moreover, the anti-angiogenesis effect of AZD6244 may predominantly attribute to its modulation on VEGF through p-ERK − c-Fos − HIF-1α integrated signal pathways. In conclusions, High p-ERK expression was associated with advanced TNM stage, increased lymphovascular invasion and poor survival. Targeting inhibition of p-ERK by AZD6244 suppress gastric cancer xenografts by blockage of angiogenesis without systemic toxicity. The anti-angiogenesis effect afford by AZD6244 may attribute to its modulation on p-ERK − c-Fos − HIF-1α − VEGF integrated signal pathways.

Gastric cancer is the fifth most common malignancy but the third leading cause of cancer-related deaths worldwide^1^. Although the combination of 5-fluorouracil and platinum analog is the most commonly accepted therapy worldwide, the prognosis remains poor^2^. Thus, there is a substantial need for other therapeutic agents that may offer superior efficacy for patients suffering from gastric cancer.

Tumors exploit various pathways to recruit blood vessel for it is indispensable for tumor growth and metastasis^3^,^4^. Angiogenesis, the development of new blood vessels from the existing vasculature, has become the most widely investigated mode of new vessel formation in gastric cancer. Inhibition of angiogenesis remarkably ameliorates gastric cancer, which suggesting the potential role of anti-angiogenesis strategy in the treatment of gastric cancer^5^,^6^,^7^.

The mitogen-activated protein kinase (MAPK) signaling cascades-the extracellular signal-related kinase (ERK) pathway is important for proliferation, differentiation, apoptosis and angiogenesis^8^. It has been demonstrated in previous studies that the MAPK-ERK pathway is overactive in gastric cancer, and its activation is associated with angiogenesis^9^. Consequently, targeting inhibition of the MEK-ERK pathway may offer an effective treatment for gastric cancer. AZD6244 (ARRY-142886) is a highly selective MAPK-ERK kinase (MEK) inhibitor. Regardless of the fact that AZD6244 has shown excellent clinical efficacy in a number of tumors including melanoma, biliary cancer, colorectal cancer and pancreatic cancer^10^, the anti-tumor and anti-angiogenesis efficacy of AZD6244 on gastric cancer has not been well characterized.

In the present study, we aimed to explore the anti-tumor and anti-angiogenesis effects of AZD6244 in gastric cancer. The correlation of p-ERK expression and progression to metastasis in human gastric adenocarcinoma was investigated. The anti-proliferative and apoptosis induction property of AZD6244 was investigated in human gastric cancer cells (SGC790 and BGC823) *in vitro*. The anti-tumor and anti-angiogenesis effects of AZD6244 were evaluated *in vivo* by using SGC7901 gastric cancer xenografts. Moreover, the potential mechanism afford by AZD6244 was also investigated.

## Results

### Patient characteristics

As showed in Table 1, the tissue microarray consisted of 90 patients with diagnosed gastric adenocarcinoma (53 men and 37 women, mean age 62.1 ± 12.3 years). The TNM classification of malignant tumors was applied to determine the clinicopathological stage, 9 (10%) as stage I, 27 (30%) as stage II, 50 (55.6%) as stage III, 4 (4.4%) as stage IV. The average tumor size was 5.7 ± 2.7 cm, with 35 (38.9%) tumors <5 cm and 55 (61.1%) tumors ≥ 5 cm. The lymphovascular invasion and distant metastasis were 25 (27.8%) and 4 (4.4%) of 90 patients, respectively. The five-year survival rate and median five-year survival was 36.7% and 24 months (range from 1 to 60 months), respectively.

### Correlation of phosphorylated ERK (p-ERK) expression with progression to metastasis in human gastric adenocarcinoma

**Figure 1:**
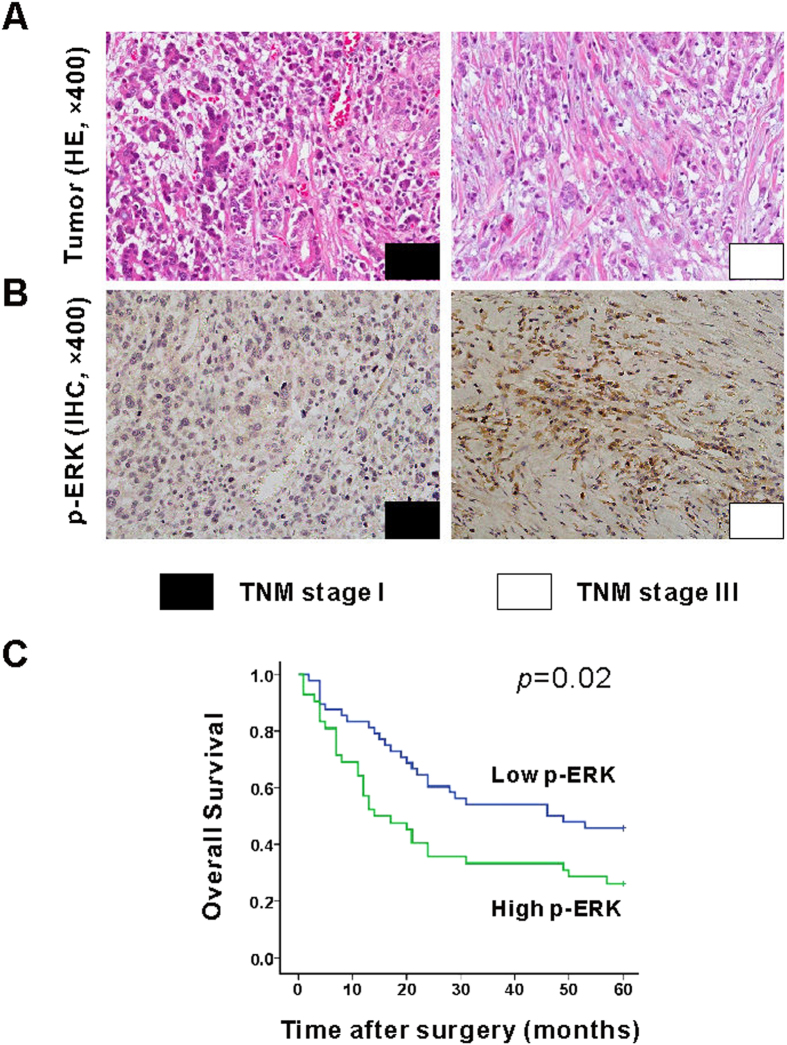
Associations of the p-ERK expression between the clinicopathological stage and overall survival in gastric adenocarcinoma tissues. Representing image for HE and IHC of p-ERK for gastric adenocarcinoma tissues was shown (**A**,**B**). The 5-years overall survival was better in patients with low p-ERK expression when compared with those with high p-ERK expression (B, *p* = 0.02, n = 90).

Positive staining of p-ERK was localized to cytoplasm and nucleus (Fig. 1B). Of all tissues, 48 tissues (53.3%) were characterized as low p-ERK expression, and 42 tissues (46.7%) were characterized as high p-ERK expression. Associations between the various clinicopathological factors and the expression of p-ERK in gastric adenocarcinoma tissues were analyzed (Table 1). A significant association between p-ERK expression and TNM stage, lymphovascular invasion was observed (*p* = 0.038 and *p* = 0.041, respectively). The rate of patients with TNM stage I∼II was higher in low p-ERK expression group when compared with that in high p-ERK expression group (50% *vs*. 28.6%). Whereas, the rate of patients with TNM stage III∼IV was lower in low p-ERK expression group when compared with that in high p-ERK expression group (50% *vs*. 71.4%). Furthermore, patients with low p-ERK expression in tumor tissues were probably with the lower risk of lymphovascular invasion when compared with that with high p-ERK expression patient (18.8% *vs*. 38.1%, *p* = 0.041). However, there was no dramatically correlation between p-ERK expression and age, gender, tumor size and distant metastasis (*p* > 0.05). Moreover, significant association between p-ERK expression and 5-years survival rate in human gastric adenocarcinoma was also established (Fig. 1B, *p* = 0.02). The 5-years survival rate in low p-ERK expression group was much higher than that in high p-ERK expression group (45.8% *vs*. 26.2%). Median 5-years survival of low and high expression group was 46 (range 2 to 60 months) and 14 (range 1 to 60 months), respectively.

### Inhibition of gastric cancer cells proliferation by AZD6244

**Figure 2:**
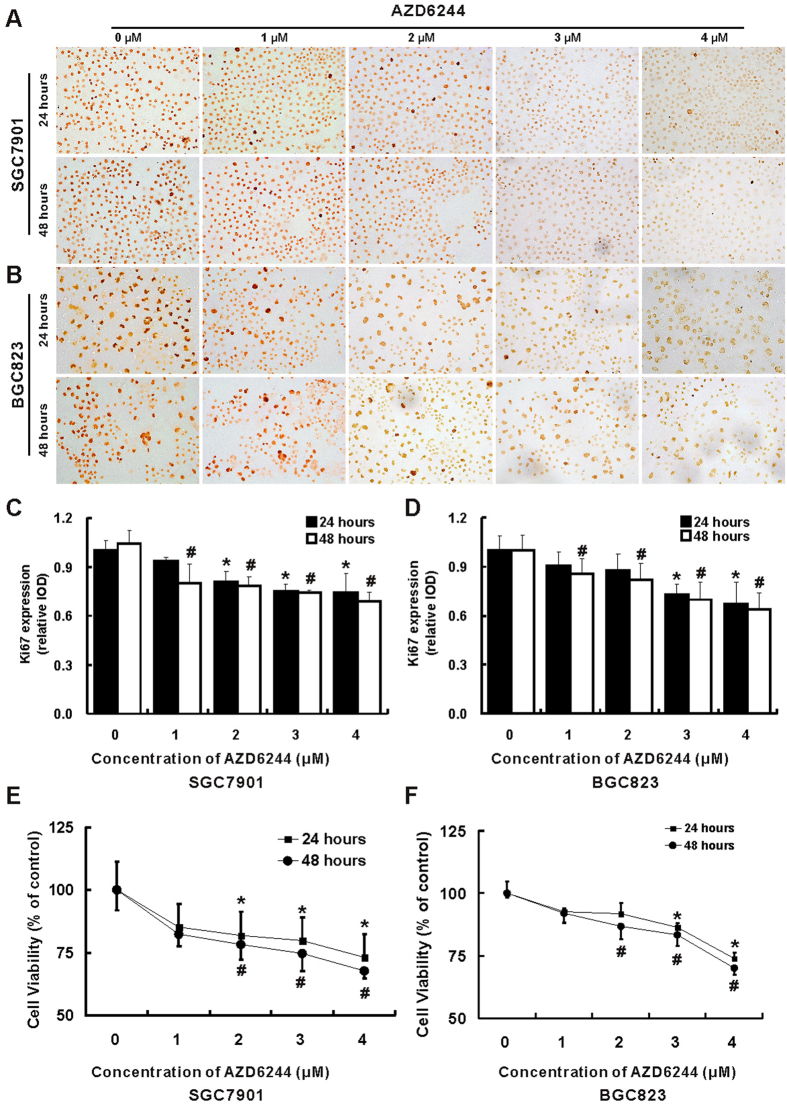
AZD6244 inhibits proliferation of gastric cancer cells. The proliferation of gastric cancer cell SGC7901 (**A**) and BGC823 (**B**) was determined by IHC of Ki67 (magnification: ~ × 400). Compared with vehicle treated SGC7901 cells, inhibition of proliferation was observed in cells treatment with high concentration of AZD6244 (2, 3 and 4 μM) for 24 hours and all given concentration of AZD6244 (1, 2, 3 and 4 μM) for 48 hours (**C**). Compared with vehicle treated BGC823 cells, proliferation was suppressed by treatment with high concentration of AZD6244 (3 and 4 μM) for 24 hours and all given concentration of AZD6244 for 48 hours (**D**). Meanwhile, the CCK-8 assay was carried out to evaluation of cell viability. Relatively high concentration of AZD6244 (2, 3 and 4 μM) was able to suppress cell viability of SGC7901 for 24 and 48 hours. Similarly, reduction of cell viability was observed in cells treatment with high concentration of AZD6244 (3 and 4 μM) for 24 and 48 hours. Furthermore, treatment with AZD6244 at the concentration of 2 μM for 48 hours was also able to inhibit cell viability. *p < 0.05 *vs*. vehicle treated cells for 24 hours; ^#^*p* < 0.05 *vs*. vehicle treated cells for 48 hours.

Treatment with AZD6244 led to both dose and time dependent inhibition of proliferation in SGC7901 and BGC823 cells (Fig. 2A–D). Treatment with AZD6244 in concentration of 1 μM and 2 μM for 24 hours was insufficient to inhibit the proliferation of BGC823, however, the proliferation of BGC823 was significant repressed by treatment with AZD6244 in concentration of 3.0 μM and 4.0 μM. In contrast, treatment with all given concentration (1, 2, 3 and 4 μM) of AZD6244 was adequately to inhibit the proliferation of BGC823 for 48 hours and SGC7901 for 24 and 48 hours. Treatment with AZD6244 at a concentration of 4.0 μM for 48 hours resulted in 31.2% and 36.2% inhibition of cell proliferation for SGC7901 and BGC823 cells, respectively. Similar results were obtained in the CCK-8 cell viability assay for SGC7901 and BGC823 cells (Fig. 2E,F).

### Promotion of gastric cancer cells apoptosis by AZD6244

**Figure 3:**
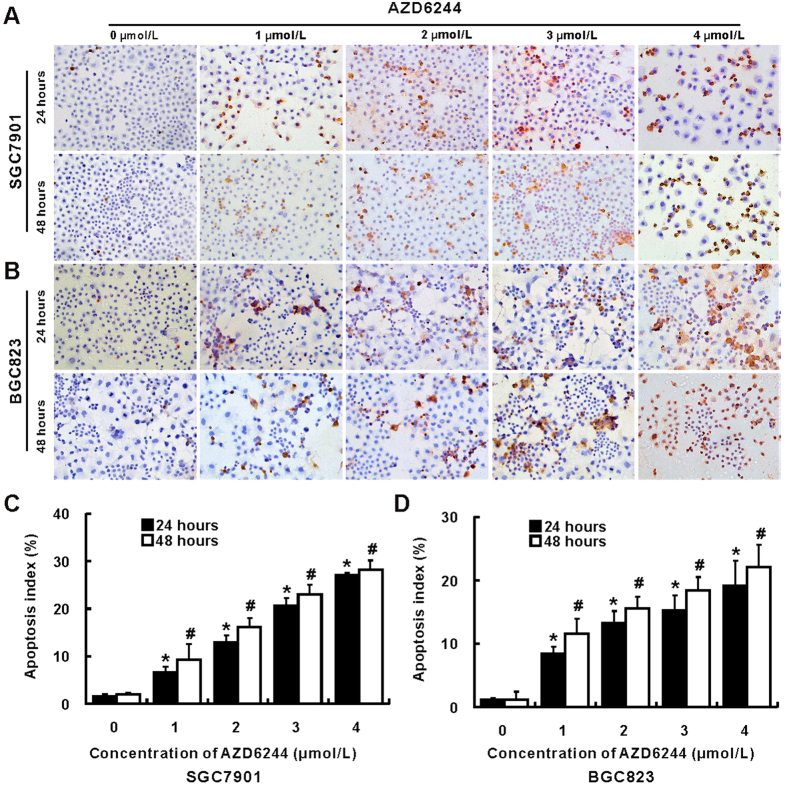
AZD6244 induces cell apoptosis of gastric cancer cells. The apoptosis of gastric cancer cell SGC7901 (**A**) and BGC823 (**B**) was determined by TUNEL (magnification: ~ × 400). Apoptosis was almost not found in vehicle treated SGC7901 cells, however, apoptotic cells were increased after treatment with all given concentration of AZD6244 (1, 2, 3 and 4 μM) for 24 and 48 hours (**C**). Few apoptotic cells were observed in vehicle treated BGC823 cells, whereas, apoptosis was enhanced after treatment with all given concentration of AZD6244 for 24 and 48 hours (**D**). The apoptosis index was referring to percentage of apoptotic cells in all cells. *p < 0.05 *vs*. vehicle treated cells for 24 hours; ^#^*p* < 0.05 *vs*. vehicle treated cells for 48 hours.

Treatment with AZD6244 induced both dose and time dependent apoptosis in SGC7901 and BGC823 cells (Fig. 3). Dramatically increased apoptosis was observed in SGC7901 and BGC823 cells after treatment with all given concentration of AZD6244 for 24 and 48 hours. Impressively, the maximal apoptosis index of SGC7901 and BGC823 was 28.2% and 22.1%, respectively, which was obtained from cells treated with AZD6244 in the concentration of 4 μM for 48 hours.

### Inhibition of ERK phosphorylation by AZD6244 in gastric cancer cells

**Figure 4:**
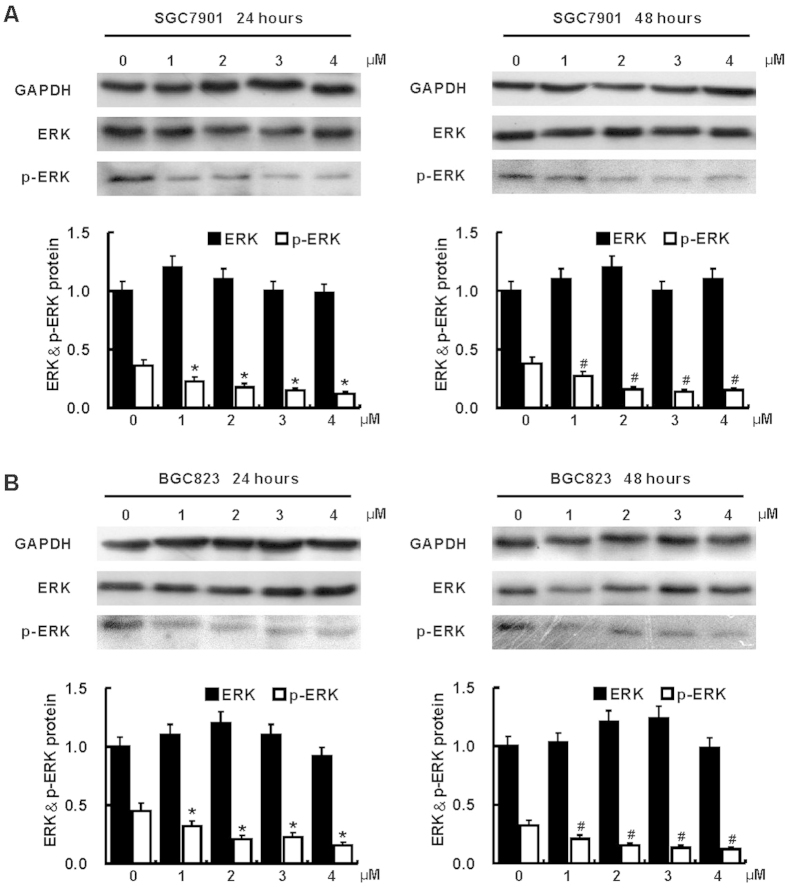
AZD6244 suppresses phosphorylation of ERK in gastric cancer cells. Phosphorylation of ERK in gastric cancer cell SGC7901 (**A**) and BGC823 (**B**) was determined by Western blot. Compared with vehicle treated SGC7901 cells, suppression of ERK phosphorylation was observed in cells treatment with all concentration of AZD6244 (1, 2, 3 and 4 μM) for 24 and 48 hours. Compared with vehicle treated BGC823 cells, ERK phosphorylation was suppressed by treatment with all given concentration of AZD6244 (1, 2, 3 and 4 μM) for 24 and 48 hours. *p < 0.05 *vs*. vehicle treated cells for 24 hours; ^#^*p* < 0.05 *vs*. vehicle treated cells for 48 hours.

Total ERK in both SGC7901 and BGC823 were comparable among all treatments; however, p-ERK was reduced after treatment with all given concentration (1, 2, 3 and 4 μM) of AZD6244 in BGC823 and SGC7901 for 24 and 48 hours when compared with corresponding vehicle treated cells (Fig. 4).

### Absence of NRAS, KRAS and BRAF mutations in gastric cancer cell lines

NRAS, KRAS and BRAF mutations were not observed in SGC7901 or BGC823 gastric cancer cell lines (Supporting Fig. 1A, B, Supporting Table 2).

### Suppression of tumor growth in SGC7901 xenografts

**Figure 5:**
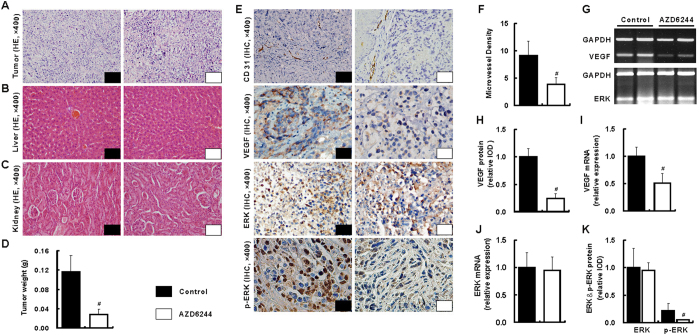
AZD6244 reduces tumor growth and angiogenesis without hepatic and renal toxicity in SGC7901 xenografts. The degree of differentiation was comparable between two groups; however, increased necrosis of tumor was visualized in the AZD6244 group (**A**). No significant pathologic change was observed in liver (**B**) and kidney (**C**). The tumor weight was significantly decreased after treatment with AZD6244 at a dosage of 50 mg/kg/day when compared to that treatment with vehicle (**D**). ERK were comparable between two groups, and much more positive staining of CD 31, VEGF and p-ERK (**E**) visualized by IHC was observed in the control group when compared with that in AZD6244 group. Consistently, the MVD (**F**), mRNA (**G,I**) and protein (**H**) of VEGF were remarkably reduced after treatment with AZD6244 at a dosage of 50 mg/kg/day. Both mRNA and protein of ERK were comparable between two groups (**J,K**); however, the p-ERK was dramatically reduced in AZD6244 group when compared with that in control group (**G**). ^#^*p* < 0.05 *vs*. control group.

All SGC7901 xenografts grew well *in situ* and appeared as nodular masses. Despite comparable in degree of differentiation between two groups, increased necrosis of tumor was visualized in the AZD6244 group (Fig. 5A). Moreover, tumor weight was significantly reduced by 75.9% in AZD6244 group when compared to that in control group (*p* = 0.016, Fig. 5D).

### Safety of AZD6244 on SGC7901 xenografts

One mouse died in the control group while no death was observed in AZD6244 group. The average body weights were comparable at the end of treatment between control and AZD6224 groups (22.3 ± 2.8 g *vs*. 21.0 ± 2.4 g, *p* = 0.744). The mice in AZD6244 group did not exhibit any physical sign of toxicity. And no toxic pathologic changes were revealed in liver and kidney (Fig. 4B,C). Furthermore, no significant differences in the serum levels of ALT, AST, total protein, albumin, bilirubin, urea and creatinine were found between two groups (Supporting Table 3).

### Reduction of angiogenesis by AZD6244 in SGC7901 xenografts

Compared with that in control group, treatment with AZD6244 induced 58.2% reduction of microvessel density (MVD) (9.1 ± 2.7 *vs.* 3.8 ± 1.3; *p < *0.001 Fig. 5E,F). Furthermore, remarkably down-regulation of vascular endothelial growth factor (VEGF) mRNA and protein were also observed in AZD6244 group when compared to that in control group (Fig. 5E,G–I).

### Inhibition of the integrated signal pathways by AZD6244 in SGC7901 xenografts

Protein and mRNA of ERK in tumors were comparable between two groups; however, p-ERK was reduced in AZD6244 group when compared with that in control group (Fig. 5E,G,J,K). Furthermore, c-Fos – c-Jun – hypoxia inducible factor 1 alpha subunit (HIF-1α), a link between ERK and VEGF, was greatly reduced after treatment with AZD6244. However, no significant inhibitory of VEGF receptor-2 (VEGFR-2) mRNA was observed after treatment with AZD6244 (Supporting Fig. 2).

### Suppression of angiogenesis by AZD6244 in human umbilical vein endothelial cell (HUVEC)

**Figure 6:**
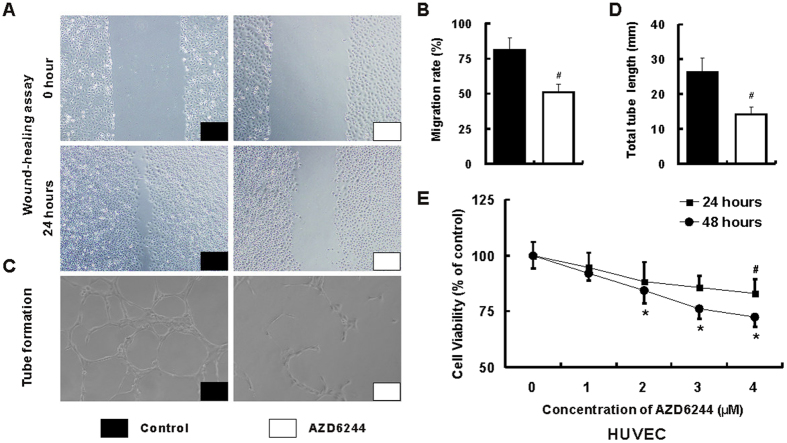
AZD6244 inhibits migration rate, tube formation and cell viability in HUVEC. The migration rate (**A,B**) and tube length (**C,D**) were remarkably reduced in AZD6244 (4 μM) treated cells when compared with that in control group. The CCK-8 assay was applied to determine cell viability of HUVEC (**E**). Inhibition of cell viability was observed in cells treatment with high concentration of AZD6244 for 24 hours (4 μM) and 48 hours (2, 3 and 4 μM) (**A**). ^#^*p* < 0.05 *vs*. control group.

Compared with vehicle treated HUVEC cells, the migration rate was markedly reduced in AZD6244 treated cells (80.8 ± 9.7% *vs*. 50.9 ± 6.1%, p < 0.001 Fig. 6A,B). Furthermore, the tube length in AZD6244 treated cells was also substantially decreased when compared with that in vehicle treated cells (26.3 ± 3.2 mm vs. 14.2 ± 1.7 mm, p < 0.001 Fig. 6C,D). Interestingly, cell viability was suppressed in HUVEC cells treatment with high concentration of AZD6244 for 24 hours (4 μM) and 48 hours (2, 3 and 4 μM) (Fig. 6E).

### Inhibition of angiogenesis through p-ERK VEGF by AZD6244

**Figure 7:**
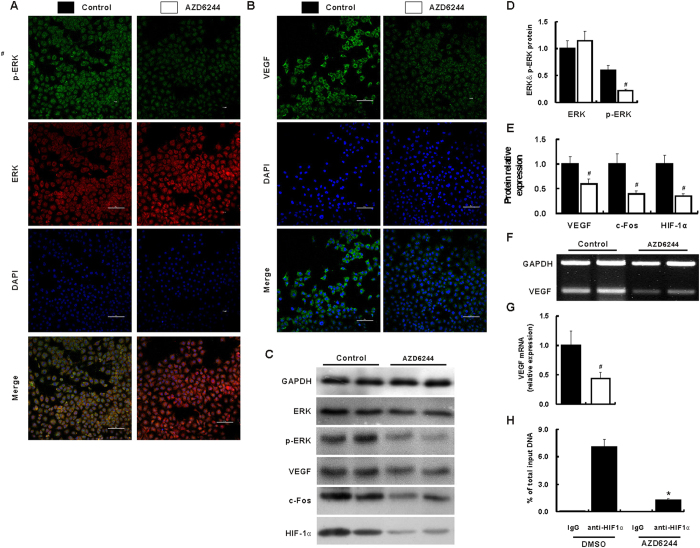
AZD6244 suppresses the p-ERK − c-FOS − HIF-1α − VEGF integrated signal pathways in HUVEC cells. HUVEC cells were treated with AZD6244 (4 μM) for 24 hours. ERK was comparable between treated treatments; however, compared with that in control group, p-ERK measured by immunocytofluorescence staining (**A**) and Western blot (**C,D**) was significantly suppressed in AZD6244 treated cells. VEGF protein and mRNA quantified by immunocytofluorescence staining (**B**), Western blot (**C,E**) and RT-PCR (**F,G**) were remarkably reduced in AZD6244 treated cells when compared with that in control group. Similarly, the protein level of c-FOS and HIF-1α (**C,E**) was also reduced after treatment with AZD6244. The effect of AZD6244 on HIF-1α binding to VEGF promoter region was analyzed by ChIP followed with qRT-PCR (**H**). IgG treated protein-DNA complexes was almost negative for PCR amplifications of the VEGF promoter. However, binging of HIF-1α to VEGF promoter was observed in mouse anti-HIF-1α antibody incubated protein-DNA complexes. Interesting, binging of HIF-1α to VEGF promoter was significantly reduced after AZD6244 treatment when compared with DMSO treated HUVEC cells (**H**). ^#^*p* < 0.05 vs. control group.

The ERK protein was comparable between two treatments; however, the p-ERK was significantly reduced after treatment with AZD6244 (Fig. 7A). These results were further confirmed by Western blot (Fig. 7C,D). Interestingly, both protein and mRNA level of VEGF was remarkably down-regulated after treatment with AZD6244 when compared with vehicle treated cells (Fig. 7B,C,E–G). Furthermore, c-Fos and HIF-1α were also significantly suppressed after treatment with AZD6244 in HUVEC cells (Fig. 7C,E). Similarly, the protein of VEGF was inhibited by AZD6244 in SGC7901 and BGC823 cells (Supporting Fig. 4).

### AZD6244 prevents HIF-1α binding within VEGF promoter

To determine whether reduced HIF-1α level prevents its associating with the VEGF promoter *in vitro*, chromatin immunoprecipitation (ChIP) assays were utilized. From the purified DNA, a 146 bp product on VEGF promoter region −1041 to −835 was amplified by qPCR for 40 cycles. In mouse IgG negative control, almost no detectable HIF-1α was associated with the VEGF promoter in DMSO and AZD6244 treated HUVEC cells (0.08 ± 0.01% and 0% of input DNA, respectively). A considerable of HIF-1α associating with the VEGF promoter was observed after mouse anti- HIF-1α was implied in DMSO treated cells (7.06 ± 0.87%), however, this association was remarkably reduced after treatment with AZD6244 (1.27 ± 0.12, *p* < 0.001 Fig. 7H).

## Discussion

Despite significant advances in systemic therapy for gastric cancer have been achieved, the prognosis remains unfavorable. Novel agents for gastric cancer therapy are urgently needed. As a highly selective inhibitor of MEK1/2 kinases that targeting inhibition of ERK phosphorylation, promising efficacy of AZD6244 for many tumors has been shown in phase II clinical trails^11^,^12^,^13^,^14^,^15^. However, the effect of AZD6244 on gastric cancer remains unclear. It was revealed in the present study that high p-ERK expression was predicted an advanced TNM stage, increased lymphovascular invasion and poor 5-years survival. *In vitro*, both dosage and time dependent anti-proliferation and pro-apoptosis of AZD6244 have been demonstrated in SGC7901 and BGC823 gastric cancer cells. Inhibition of tumor growth and angiogenesis was also revealed in SGC7901 xenografts *in vivo*. Furthermore, we provide *in vivo* and *in vitro* evidence that AZD6244 exerts anti-angiogenesis effect through p-ERK – c-Fos – HIF-1α – VEGF integrated signaling pathways.

Although numerous studies have focused on the MAPK-ERK signaling pathway in gastric cancer, few report has manifested the correlation of p-ERK expression with progression to metastasis in human gastric cancer. In the present study, a tissue microarray was employed to provide crucial clinical information for understanding gastric cancer progression better. It was suggested that patients with high p-ERK expression had significantly higher TNM stage, increased lymphovascular invasion risk and shorter survival than patients with low p-ERK expression. These finding characterize that tumor progression and invasion mediated by the ERK signaling pathway is a common pathway in gastric cancer. Furthermore, p-ERK might be a potential biomarker for progression, metastasis and survival of gastric adenocarcinoma.

Mangy mutated genes, such as p53, PI3K, and FAT4 were displayed in gastric cancer instead of genes in RAS – RAF – MEK – ERK integrated signal pathway^16^,^17^,^18^. Consistently, neither KRAS, NRAS nor BRAF mutations was observed in SGC7901 or BGC823. Once KRAS, NRAS or BRAF are mutated, it induces constitutive ERK signaling through hyperactivation of the RAS – MEK – ERK pathway which stimulating proliferation, survival and transformation of cells^19^. Thus, targeting inhibition of MAPK-ERK signal pathways in cells that harboring KRAS, NRAS or BRAF mutation could suppress tumor growth^20^. As established in previous studies, majority of tumor cell lines possess a mutation in either KRAS, NRAS or BRAF genes was sensitive to AZD6244 *in vitro* (IC50 < 1 μM). Moreover, compare with that wild-type cells or RAS mutation cells, cells harboring BRAF V600E mutation are associated with enhanced sensitivity to MEK inhibitors^20^. However, in the present study, AZD6244 did not achieve 50% inhibition of SGC7901 and BGC823 cell proliferation and cell viability even at the concentration of 4 μM for their absence of KRAS, NRAS and BRAF mutation. Since apoptosis cells were remarkably increased by treatment with all given concentration of AZD6244, the maximal apoptosis index was 28.2% and 22.1% for SGC7901 and BGC823, respectively. These results suggested that SGC7901 and BGC823 gastric cancer cells were relative resistance to AZD6244 *in vitro*. And such resistance was not attributed to the insufficient inhibition of ERK phosphorylation, because AZD6244 was able to reduce p-ERK expression in all given concentrations.

Contrary to these *in vitro* results, tumor growth was reduced by 75.9% at a dosage of 50 mg/kg. Efficiency of AZD6244 on SGC7901 xenografts growth is equal to tumors harboring BRAF mutation at some dosage, such as, HT-29 colorectal tumor xenografts (70%)^21^. This discrepancy between resistance *in vitro* and efficacy *in vivo* has been also revealed in breast cancer (Zr-75-1) and pancreatic cancer (PANC-1 and BxPC3)^21^. However, the mechanism remains largely unknown. It is evident in previous studies that the MAPK-ERK pathway is overactive in gastric cancer, and its activation is associated with angiogenesis^9^. Moreover, inhibition of tumor angiogenesis effectively suppresses tumor growth and metastasis^22^. In the present study, high p-ERK expression was predicting an advanced TNM stage, increased lymphovascular invasion and poor 5-years survival. Treatment with AZD6244 results in 58.2% reduction of MVD in SGC7901 xenografts, as well as by ~47% suppression of tube formation and migration rate in HUVEC cells. Consequently, it could be presumed that the efficacy observed in the SGC7901 xenografts may attribute to inhibition of angiogenesis *in vivo*.

More than 100 targets have been verified as substrate of ERK1/2^23^. Among the numerous substrates, VEGF has been identified as a key mediator of angiogenesis. In the present study, p-ERK and VEGF were remarkably reduced after blockage of MEK1/2 kinases with AZD6244 both *in vivo* and *in vitro*. In accordance with this finding, another MEK1/2 inhibitor U0126 was able to decrease the expression of HIF-1α protein and the paracrine secretion of VEGF in SGC7901 gastric cancer cells^24^. Consequently, AZD6244 may reduce both gastric cancer and endothelial VEGF expression to suppress angiogenesis via ERK signaling pathway. Of the integrated signal pathways linking p-ERK and VEGF, c-Fos and HIF-1α were down-regulated by AZD6244 both *in vitro* and *in vivo*. The c-Fos could dimerise with c-Jun to form the AP-1 transcription factor, which regulates transcription of a diverse range of genes^25^,^26^. HIF-1α is generally considered as one of the potential transcription factor-binding sites for VEGF. AZD6244 could prevent HIF-1α binding to VEGF promoter and hereby decrease VEGF mRNA and protein expression in HUVEC. Accordingly, the anti-angiogenesis effect of AZD6244 may predominantly attribute to its modulation on VEGF through the down-regulation of integrated signal pathways involving p-ERK − c-Fos − HIF-1α − VEGF. However, it has yet to be verified whether other molecular also involves in the anti-angiogenesis effect afford by AZD6244.

The highly selective MEK inhibitor AZD6244 has shown excellent clinical efficacy in several tumors, particularly in tumors harboring KRAS or BRAF mutations^13^,^27^. However, BRAF mutations were almost not found in gastric cancer tissues and gastric cancer cell lines^18^. Despite of minimal effect on proliferation and cell viability, AZD6244 inhibits tumor growth by blockage of angiogenesis without systemic toxicity in the present study. In consistent with our results, superior efficacy has also been found in various tumors that absence of BRAF mutations after treated with MEK inhibitors . Thus, treatment of gastric cancer with AZD6244 may offer a potential therapy for patients suffering from gastric cancer. Several angiogenesis inhibitors, such as bevacizumab, sorafenib, ramucirumab, sunitinib, have been approved or tested in the treatment of gastric cancer^5^,^6^,^7^,^28^,^29^. Sorafenib treatment in gastric cancer xenografts leads to a RAF/ERK feedback loop, whereas, suppression of this feedback loop by AZD6244 cloud enhance the anti-tumor and anti-angiogenesis effect of sorafenib in gastric cancer xenografts, whereas AZD6244 alone had a modest effect on anti-tumor and anti-angiogenesis^9^. The differences in experimental design between Yang^9^ and this study may contribute to the conflicting results in terms of 1) the different gastric cancer xenografts of GC-28-1107 and GC-27-0208 flanks xenografts *vs*. SGC7901 gastric xenograft. 2) AZD6244 dosage of 25 mg/kg BW daily *vs*. 50 mg/kg BW daily.

## Conclusions

High p-ERK expression was associated with advanced TNM stage, increased lymphovascular invasion and poor survival. Despite of minimal effect on cell viability, AZD6244 inhibited tumor growth of SGC7901 xenografts by blockage of angiogenesis without systemic toxicity. The anti-angiogenesis effect afford by AZD6244 may attribute to its modulation on VEGF through the down-regulation of integrated signal pathways involving p-ERK − c-Fos − HIF-1α − VEGF. Anti-angiogenesis treatment of gastric cancer with AZD6244 may offer a highly valuable approach for patients suffering from gastric cancer.

## Materials and Methods

The animal experiment was approved and conducted according to the regulations set by the Animal Use and Care Committee of Sichuan University. And all experiments were carried out in accordance with the manufacturer’s instructions.

### Tissue microarray and Immunohistochemistry (IHC)

The gastric adenocarcinoma tissue microarray implied in the present study was obtained from National Engineering Center for Biochip at Shanghai (Shanghai, China). Gastric adenocarcinoma tissues were obtained from 90 gastric cancer patients undergoing complete surgical resection of the gastric tumor between August 2008 and March 2009. All patients were followed up until April 2014. Detailed clinical and pathologic information of patients was displayed in Table 1.

For IHC, tissue microarray were deparaffinized in xylene and serial ethanol dilutions. Antigen retrieval was performed by heating the tissue microarray in 10 mM sodium citrate buffer. After blocking of endogenous peroxidase by H_2_O_2_, the tissue microarray was incubated with rabbit anti- p-ERK (1:150, Santa Cruz, Santa Cruz, CA, USA) overnight at 4°C followed by incubation with horseradish-peroxidase (HRP) -conjugated secondary antibody kits. The tissue microarray was then stained with a solution of 3, 3-diaminobenzidine tetrahydrochloride and counterstained with haematoxylin. Afterwards, the tissue microarray was assessed by two pathologists who were blinded to clinical and pathologic information. The staining intensity of p-ERK was determined as previous described^30^. Accordingly, the expression of p-ERK was divided into low p-ERK (score 0–3) and high p-ERK (score 3.1–8) group.

### Cell culture and treatments

HUVEC, human gastric cancer cell SGC7901 and BGC823 were obtained from the Type Culture Collection of the Chinese Academy of Sciences (Shanghai, China). Cells were cultured in Dulbecco’s modified Eagle’s medium (HyClone, Logan, UT, USA) or RPMI-1640 (HyClone) containing 10% fetal bovine serum (FBS, Gibco, Logan, UT, USA) and 100 U penicillin and streptomycin at 37 °C in a humidified atmosphere containing 5% CO_2_. All cells were serum-starved with 0.5% FBS 24 hours before incubated with corresponding treatments.

### CCK8 for cell viability

Cell viability was determined by using CCK8 (Dojindo, Kumamoto, Japan) according to the instruction of manufacture. Cells were seeded in 96-well plates for 24 hours. Then cells were treated with serials concentration (0, 1, 2, 3 and 4 μM) of AZD6244 (AstraZeneca, London, UK) for 24 or 48 hours. A 10 μL of CCK8 solution was then added to each well, and the plates were incubated at 37°C for 2 hours. The optical density (OD) of each well was measured at 450 nm.

### IHC assay for proliferation

SGC7901 and BGC823 cells in the logarithmic growth phase were cultured on coverslips at the bottom of 24-well plates. Cells were treated with serials concentration (0, 1, 2, 3 and 4 μM) of AZD6244 for 24 or 48 hours. The cells were hereby fixed with 4% paraformaldehyde before permeabilization with 0.1% Triton X-100. After blocking with 1% goat serum, cells were incubated with Ki67 primary antibody (1:200, Santa Cruz) overnight at 4°C followed by incubation with HRP-conjugated secondary antibody kits. The average integrated optical density (IOD) was calculated with Image-Pro plus 6.0 software (Media Cybernetics, Silver Spring, MD, USA).

### TUNEL for apoptosis

At the end of corresponding treatments, apoptosis was detected by the terminal deoxynucleotidyl transferase-mediated dUTP nick-end labelling (TUNEL) assay kit (Roche Diagnostics, Mannheim, Germany) according to the manufacturer’s instructions. The cells were counterstained with hematoxylin. For quantification of apoptosis index, five random fields at × 400 magnifications were captured and the percentages of positive cells were calculated for each section.

### Mutational analysis

Genomic DNA was extracted from SGC7901 and BGC823 cells by using TIANamp genomic DNA kit (Tiangen, Beijing, China). Polymerase chain reaction (PCR) was performed and PCR products were bidirectionally sequenced (Invitrogen, Carlsbad, CA, USA). Primers are listed in supporting Table 1.

### Human gastric cancer xenografts

Eighteen healthy male BAL b /c nude mice, weighing 18–23 g, were purchased from the Experimental Animal Center of Sichuan University (Chengdu, China). The mice were kept under 12 hours’ light-dark cycles at a constant temperature and humidity with free access to chow and water. Briefly, 1 × 10^7^ of SGC7901 cells were subcutaneously injected into the left flank of 2 nude mice. The tumors were aseptically dissected and mechanically minced after 4 weeks. A piece of tumor (~2 mm^3^) was transplanted under gastric serosa of each nude mouse. Then, 16 mice were randomized into control and AZD6244 groups with 8 mice in each group. AZD6244 group received AZD6244 (50 mg/kg/day) and the control group received vehicle by gastric gavage for 30 days after 1 week of tumor transplantation. The mice were sacrificed under anesthesia at the end of experiments. Tumors were weighed and cut longitudinally to provide a representative fragment for immunohistochemistry and RNA analysis. Serum was collected and stored at −80 °C until analysis.

### Semiquantitative real-time PCR (RT-PCR)

Total RNA was extracted by using TRIzol reagent (Invitrogen). The RNA was treated with DNase to remove potential genomic contamination, and reverse-transcribed by using a first-strand cDNA kit with random hexamers (Fermentas, Burlington, Canada). For all PCR analyses, the cycles were chosen at the logarithmic level for each gene tested. An equal volume of PCR product was analyzed by 2% agarose gel electrophoresis. The gels were scanned and quantified by using Quantity One software 4. 5. 0 (Bio-Rad, Hercules, CA, USA). Primers are listed in the supporting Table 1. The mRNA expression was normalized to glyceraldehyde-3-phosphate dehydrogenase (GAPDH) and shown as fold changes to the control group.

### Histology and IHC for gastric cancer xenograft

All samples (tumor, liver and kidney) fixed in 4% paraformaldehyde were embedded and sectioned. Sections (thickness of 6 μm) were stained with hematoxylin-eosin (HE). The sections were incubated with primary antibodies (CD31, 1:100; vascular endothelial growth factor (VEGF), 1:200; ERK, 1:200; p-ERK, 1:150, Santa Cruz) overnight at 4°C followed by incubation with HRP-conjugated secondary antibody kits. The expression of proteins was calculated as average IOD. The MVD was calculated as the average number of CD31-positive vessels in five random fields at × 400 magnifications.

### Western blot analysis for protein expression

Whole proteins from cultured cells were extracted by using protein extraction kit (Nanjing Kaiji, Nanjing, China). Equal amounts of proteins (50 μg) from each sample were resolved in 8% SDS-PAGE gel and transferred to PVDF membrane (Millipore, Billerica, MA, USA). Non-specific binding sites in the membranes were blocked with 5% non-fat dry milk in incubation buffer before addition of primary antibodies (GAPDH, VEGF, c-Fos, HIF-1α, ERK and pERK, 1:500∼1:2000). Blots were washed and incubated with appropriate HRP-conjugated secondary antibodies. Protein bands were visualized by using an ECL detection kit (Amersham Pharmacia, Uppsala, Sweden). Protein expression was determined by using Quantity One software 4.5.0 and was normalized to GAPDH.

### Immunocytofluorescence staining for VEGF, ERK and p-ERK

HUVEC cells were cultured on coverslips at the bottom of 24-well plates. The cells were hereby fixed with 4% paraformaldehyde before permeabilization with 0.1% Triton X-100. After blocking with 1% goat serum, cells were incubated with primary antibodies overnight at 4°C followed by incubation with FITC-conjugated and TRITC-conjugated secondary antibodies. Cell nuclei were stained with 4′, 6-diamino-2-phenylindole (DAPI, Roche). Slides were coverslipped with anti-fading medium and visualized with a confocal microscope.

### Wound-healing model for cell migration assay

HUVEC cells were seeded at a density of 5 × 10^4^ cells/well in 6-well plates and allowed to form a confluent monolayer. Wound was created by scraping confluent cell monolayer with a 20-200 μL micropipette tip to denude a strip of monolayer ~100 μm width. Cells were then washed triple with phosphate buffered saline and immediately incubated with corresponding treatments for 24 hours. The images were taken under the inverted microscope at 0 hours and 24 hours after scratching. The average distance of the wound was calculated by using Image-Pro plus 6.0 software. The migration rate was calculated by the formula: (width_0 hours_ − width _24 hours_)/width _0 hours_ × 100%.

### Tube formation assay for angiogenesis *in vitro*

HUVEC cells were seeded at a density of 5 × 10^3^ cells/well in matrigel (BD Bioscience, Billerica, MA, USA) coated 24-well plates in the presence of corresponding treatments. Five different fields were chosen randomly in each well, and photographs were taken under the inverted microscope at 24 hours. The total length per microscopic was measured by using Image-Pro plus 6.0 software.

### Chromatin immunoprecipitation assay(ChIP) for HIF-1α

Chromatin of HUVEC cells was fixed and immunoprecipitated by using EZ Magna ChIP G kit (17–409, Millipore) and Enzymatic ChIP Kit (17–375, Millipore) according to manufacturer’s instructions. HUVEC cells were treated with DMSO or AZD6244 (4 μM) for 24 hours. Cells were fixed with 1% formaldehyde and quenched by 2 M glycine. Scraping cells from each dish after treated cells with protease inhibitor cocktail II, centrifuge the cells and collected the deposition. Resuspend cells in EZ-Zyme™ lysis buffer and centrifuge cell lysate at 2500 g for 10 minutes at 4 °C to pellet nuclei. Afterwards, pre-warmed EZ-Zyme™ Enzymatic Cocktail was added to cleave DNA into 180 bp ∼ 360 bp. The chromatin solutions were precleared and 5 μL of the supernatant was removed as input. The protein-DNA complexes were incubated with mouse anti-HIF-1α (Abcam, London, UK) or IgG (negative control, Milipore) and rotated overnight at 4 °C. Immunocomplexes were affinity absorbed with protein G magnetic beads and collected by centrifugation. Crosslinks were reversed by adding proteinase K contained ChIP elution buffer. Free DNA was purification by using spin columns. The eluates were employed as a template for qPCR with a CFX96 real-time PCR detection system (Bio-Rad). The specific primer for amplifications of VEGF promoter region from −1041 to −835 was revised according previous publication (supporting Table 1)^31^.

### Statistical analysis

All data were expressed as mean ± standard deviation and were analyzed by SPSS 13.0 software (SPSS, Chicago, IL, USA). Quantitative data were analyzed by using one-way ANOVA. Survival curves were evaluated by using the Kaplan-Meier method and compared by the log-rank test. The χ^2^ test was applied to analyze the difference of clinicopathological parameters between low and high p-ERK expression groups. A value of *p* < 0.05 was considered significant.

## Additional Information

**How to cite this article**: Gao, J.-H. *et al.* Targeting inhibition of extracellular signal-regulated kinase kinase pathway with AZD6244 (ARRY-142886) suppresses growth and angiogenesis of gastric cancer. *Sci. Rep.* **5**, 16382; doi: 10.1038/srep16382 (2015).

## Supplementary Material

Supplementary Information

## Figures and Tables

**Table 1:** Relationship between p-ERK expression and clinicopathological parameters in 90 gastric adenocarcinoma cases.

Characteristic	Number of casesnumber(%)	p-ERK expression:number(%)		P value
		Low	High	
Total	90	48(53.3)	42(46.7)	
Average years	62.1 ± 12.3	61.6 ± 11.5	62.5 ± 13.4	0.66
< 65	52(57.8)	30(62.5)	22(52.4)	0.332
≥ 65	38(42.3)	18(37.5)	20(47.6)	
Gender				
Male	53(58.9)	30(62.5)	23(54.8)	0.457
Female	37(41.1)	18(37.5)	19(45.2)	
TNM stage				
I-II	36(40)	24(50.0)	12(28.6)	0.038
III- IV	54(60)	24(50.0)	30(71.4)	
Tumor size (cm)	5.7 ± 2.7	5.3 ± 2.3	6.2 ± 2.9	0.093
< 5 cm	35(38.9)	20(41.7)	15(35.7)	0.563
≥ 5 cm	55(61.1)	28(58.3)	27(64.3)	
Lymphovascular Invasion	25(27.8)	9(18.8)	16(38.1)	0.041
Distant metastasis	4(4.4)	3(6.3)	1(2.4)	0.374
