# Author Correction: Targeting inhibition of extracellular signal-regulated kinase kinase pathway with AZD6244 (ARRY-142886) suppresses growth and angiogenesis of gastric cancer

**DOI:** 10.1038/s41598-022-07805-0

**Published:** 2022-03-01

**Authors:** Jin-Hang Gao, Chun-Hui Wang, Huan Tong, Shi-Lei Wen, Zhi-Yin Huang, Cheng-Wei Tang

**Affiliations:** 1 Division of Peptides Related With Human Diseases, State Key Laboratory of Biotherapy, West China Hospital, Sichuan University, Chengdu, China; 2 Department of Gastroenterology, West China Hospital, Sichuan University, Chengdu, China; 3 Department of Human Anatomy, Academy of Preclinical and Forensic Medicine, Sichuan University, Chengdu, China

Correction to: *Scientific Reports* 10.1038/srep16382, published online 16 November 2015

This Article contains an error in Figure 2. As a result of a mistake in Figure 2A assembly, the Ki67 expression of SGC7901 cells in AZD6244 1 μM, 2 μM and 3 μM of 48 hours treatment were actually the corresponding photos from 24 hours treatment. The correct Figure 2 and accompanying legend appear below.

**Figure 2:**
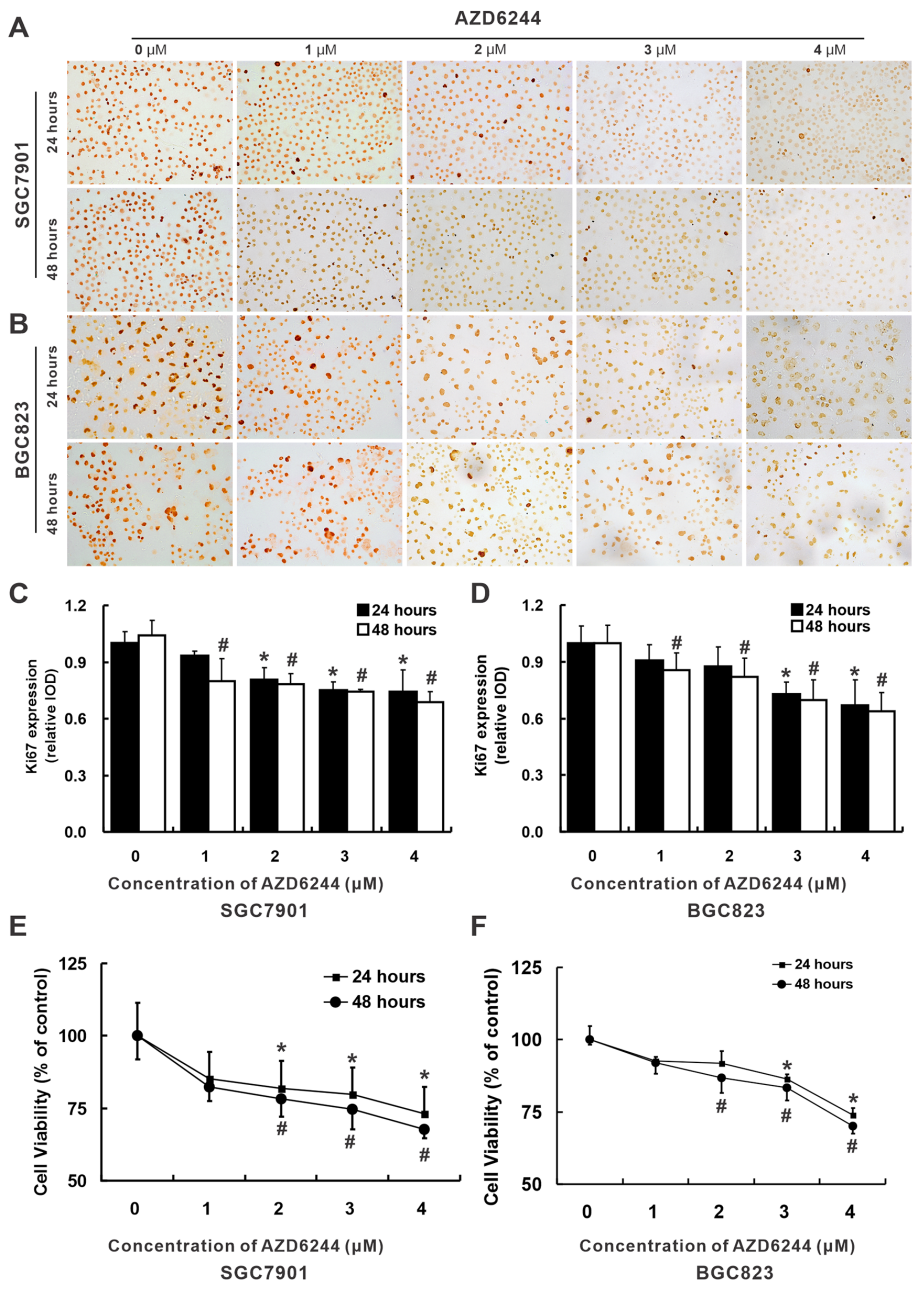
AZD6244 inhibits proliferation of gastric cancer cells. The proliferation of gastric cancer cell SGC7901 (**A**) and BGC823 (**B**) was determined by IHC of Ki67 (magnification: ~  × 400). Compared with vehicle treated SGC7901 cells, inhibition of proliferation was observed in cells treatment with high concentration of AZD6244 (2, 3 and 4 μM) for 24 hours and all given concentration of AZD6244 (1, 2, 3 and 4 μM) for 48 hours (**C**). Compared with vehicle treated BGC823 cells, proliferation was suppressed by treatment with high concentration of AZD6244 (3 and 4 μM) for 24 hours and all given concentration of AZD6244 for 48 hours (**D**). Meanwhile, the CCK-8 assay was carried out to evaluation of cell viability. Relatively high concentration of AZD6244 (2, 3 and 4 μM) was able to suppress cell viability of SGC7901 for 24 and 48 hours. Similarly, reduction of cell viability was observed in cells treatment with high concentration of AZD6244 (3 and 4 μM) for 24 and 48 hours. Furthermore, treatment with AZD6244 at the concentration of 2 μM for 48 hours was also able to inhibit cell viability. *p < 0.05 *vs*. vehicle treated cells for 24 hours; ^#^*p* < 0.05 *vs*. vehicle treated cells for 48 hours.

This change does not affect the conclusions of the Article.

