# Supplementary material for: Targeting inhibition of extracellular signal-regulated kinase kinase pathway with AZD6244 (ARRY-142886) suppresses growth and angiogenesis of gastric cancer
Source: Sci Rep. 2015 Nov 16;5:16382. doi: 10.1038/srep16382 (PMC4644956; doi:10.1038/srep16382)
Supplement: Supplementary Information [file srep16382-s1.doc]

**Targeting inhibition of extracellular signal-regulated kinase kinase pathway with AZD6244 (ARRY-142886) suppresses growth and angiogenesis of gastric cancer**

**Jin-Hang Gao**^+^**^1, 2^, Chun-Hui Wang**^+^**^2^, Huan Tong^2^, Shi-Lei Wen^3^, Zhi-Yin Huang^2^, Cheng-Wei Tang*****^1, 2^**

*1, Division of Peptides Related with Human Diseases, State Key Laboratory of Biotherapy, West China Hospital, Sichuan University, Chengdu, China.*

*2, Department of Gastroenterology, West China Hospital, Sichuan University, Chengdu, China.*

*3, Department of Human Anatomy, Academy of Preclinical and Forensic Medicine, Sichuan University, Chengdu, China.*

**Correspondence*: Cheng-Wei Tang, [shcqcdmed@163.com](mailto:shcqcdmed@163.com);

^+^Jin-Hang Gao and Chun-Hui Wang contributed equally to this study.

**Supplementary Dataset**

**Supporting table 1**

**Supporting table 1 List of primers**

| **Gene** | **GenBank Accession No.** |  | **Sequence Primers (5’-3’)** | **Expected product Size** |
| --- | --- | --- | --- | --- |
| ***BRAF***  ***exon 11*** | NC_000007.14 | F | TCCCTCTCAGGCATAAGGTAA | 313 bp |
|  |  | R | CGAACAGTGAATATTTCCTTTGAT |  |
| ***BRAF***  ***exon 15*** | NC_000007.14 | F | GGCAGAGTGCCTCAAAAAGAA | 224 bp |
|  |  | R | AACCAGCCCGATTCAAGGA |  |
| ***KRAS***  ***exon 2*** | NC_000012.12 | F | GTGTGACATGTTCTAATATAGTCA | 214 bp |
|  |  | R | GAATGGTCCTGCACCAGTAA |  |
| ***KRAS***  ***exon 3*** | NC_000012.12 | F | TCAAGTCCTTTGCCCATTTT | 375 bp |
|  |  | R | TGCATGGCATTAGCAAAGAC |  |
| ***NRAS***  ***exon 2*** | NC_000001.11 | F | CTTGCTGGTGTGAAATGACTG | 176 bp |
|  |  | R | TCCGACAAGTGAGAGACAGG |  |
| ***NRAS***  ***exon 3*** | NC_000001.11 | F | GTGAAACCTGTTTGTTGGAC | 149 bp |
|  |  | R | CCTGTAGAGGTTAATATCCG |  |
| ***c-Fos*** | [NM_005252.3](http://www.ncbi.nlm.nih.gov/nucleotide/254750707?report=genbank&log$=nucltop&blast_rank=1&RID=6ZP1UNXD01R) | F | GGAGAATCCGAAGGGAAAGG | 367 bp |
|  |  | R | ATGCTGCTGATGCTCTTGACA |  |
| ***c-Jun*** | [NM_002228.3](http://www.ncbi.nlm.nih.gov/nucleotide/44890066?report=genbank&log$=nucltop&blast_rank=1&RID=6ZP2EG4F01R) | F | GGATCAAGGCGGAGAGGAAG | 222 bp |
|  |  | R | GCGTTAGCATGAGTTG GCAC |  |
| ***ERK*** | [NM_002746.2](http://www.ncbi.nlm.nih.gov/nucleotide/91718898?report=genbank&log$=nucltop&blast_rank=4&RID=6ZP2Y4TJ01R) | F | TCAACACCACCTGCGACCTT | 102 bp |
|  |  | R | GCGTAGCCACATACTCCGTCA |  |
| ***GAPDH*** | [NM_002046.4](http://www.ncbi.nlm.nih.gov/nucleotide/378404906?report=genbank&log$=nucltop&blast_rank=2&RID=6ZP3FGCW01R) | F | TGGGTGTGAACCATGAGAAGT | 475 bp |
|  |  | R | TGGGTGTCGCTGTTGAAGTC |  |
| ***HIF-1α*** | [NM_001530.3](http://www.ncbi.nlm.nih.gov/nucleotide/194473733?report=genbank&log$=nucltop&blast_rank=3&RID=6ZP3W18G01R) | F | TTTGCTGAAGACACAGAAGCAAAGA | 171 bp |
|  |  | R | TTGAGGACTTGCGCTTTCAGG |  |
| ***VEGF*** | [NM_003376.5](http://www.ncbi.nlm.nih.gov/nucleotide/284172448?report=genbank&log$=nucltop&blast_rank=18&RID=6ZP4864001R) | F | CCTGGTGGACATCTTCCAGGA | 196 bp |
|  |  | R | GAAGCTCATCTCTCCTATGTG |  |
| ***VEGFR-2*** | [NM_002253.2](http://www.ncbi.nlm.nih.gov/nucleotide/195546779?report=genbank&log$=nucltop&blast_rank=1&RID=6ZP4KU5E01R) | F | ACCTTGGAGCATCTCATCTGT | 367 bp |
|  |  | R | TTCCTTCTTTCAATCGCCTAC |  |
| ***VEGF-ChIP*** | NC_000006.12 | F | CAGGAACAAGGGCCTCTGTCT | 146 bp |
|  |  | R | TGGAGCTGAGAACGGGAAGCT |  |

F, Forward sequence; R, Reverse sequence; GAPDH, glyceraldehyde-3-phosphate dehydrogenase; HIF-1α, hypoxia inducible factor 1, alpha subunit; VEGF, vascular endothelial growth factor; VEGFR-2, VEGF receptor 2, VEGF-ChIP, the primer for ChIP.

**Supporting Table 2**

**Supporting table 2 BRAF, K-RAS and N-RAS** mutations in cell lines

|  | **BGC823** | **SGC7901** | **HUVEC** | **HT-29** |
| --- | --- | --- | --- | --- |
| **BRAF exon 11** | N/A | N/A | N/A | N/A |
| **BRAF exon 15** | N/A | N/A | N/A | **V600E T→A** |
| **KRAS exon 2** | N/A | N/A | N/A | N/A |
| **KRAS exon 3** | N/A | N/A | N/A | N/A |
| **NRAS exon 2** | N/A | N/A | N/A | N/A |
| **NRAS exon 3** | N/A | N/A | N/A | N/A |

**Supporting Table 3**

**Supporting table 3 Effects of AZD6244 on the liver and renal functions**

|  | **Control** | **AZD6244** |
| --- | --- | --- |
| **ALT (IU/L)** | 33.9 ± 5.1 | 30.8 ±12.1 |
| **AST (IU/L)** | 210.3 ± 27.3 | 230.6 ± 43.6 |
| **Total protein (g/L)** | 51.1 ± 2.9 | 52.4 ± 6.2 |
| **Albumin (g/L)** | 27.2 ± 1.5 | 28.3 ± 5.3 |
| **Bilirubin (μM)** | 1.9 ± 0.6 | 2.1 ± 0.8 |
| **Urea (mM)** | 13.19 ± 0.82 | 14.01 ± 2.64 |
| **Creatinine (μM)** | 10.4 ± 2.1 | 11.3 ± 2.2 |

ALT, alanine aminotransferase; AST, aspartate aminotransferase.

**
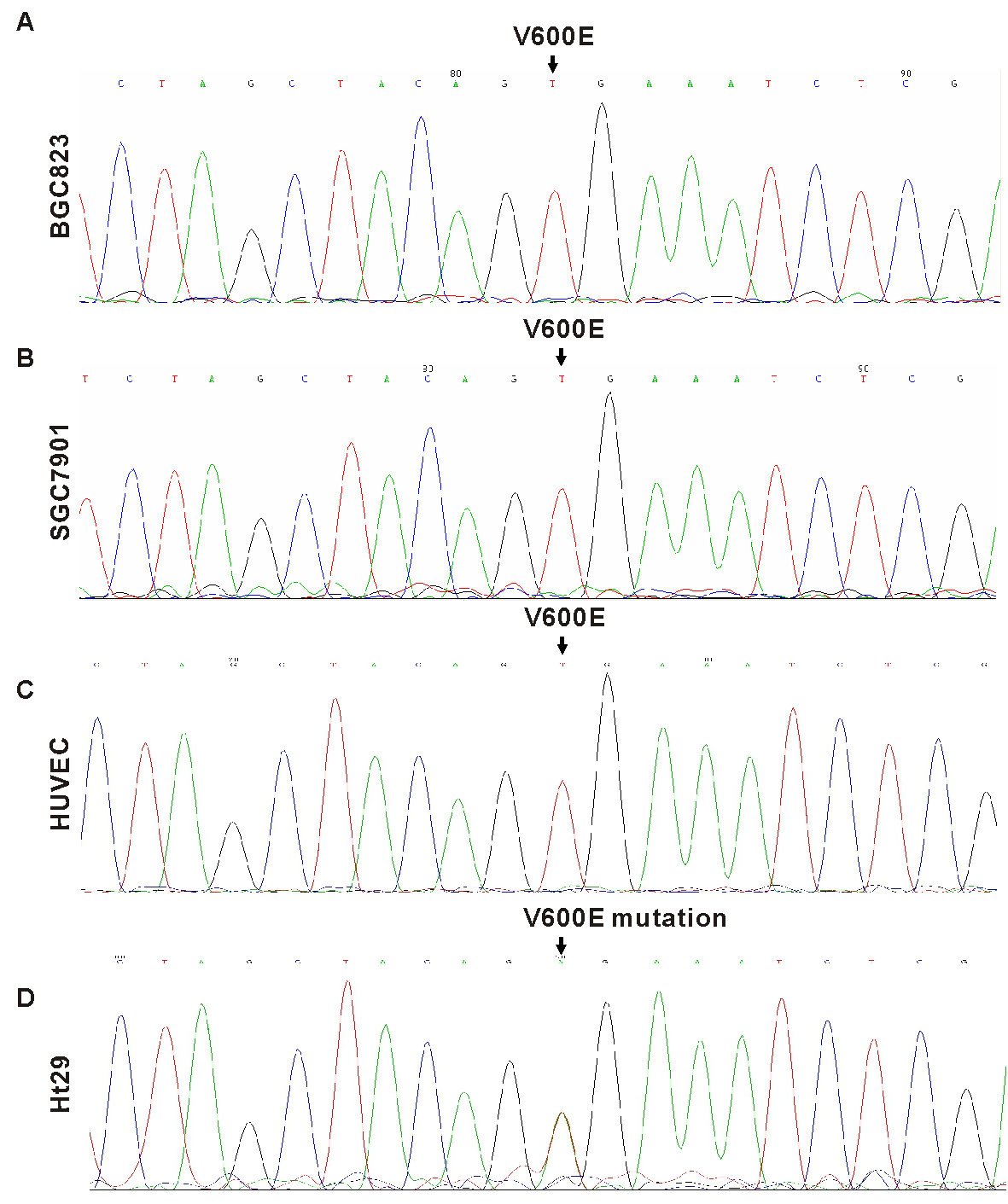
**

**Supporting figure 1 Absence of BRAF V600E mutation in cell lines**

BRAF V600E mutation is termed as T to A convert at V600E site. SGC7901 (A), BGC823 (B) and HUVEC (C) cell lines were wild-type of the BRAF. The BRAF V600E mutation was found in the positive control HT29 cell line (D).


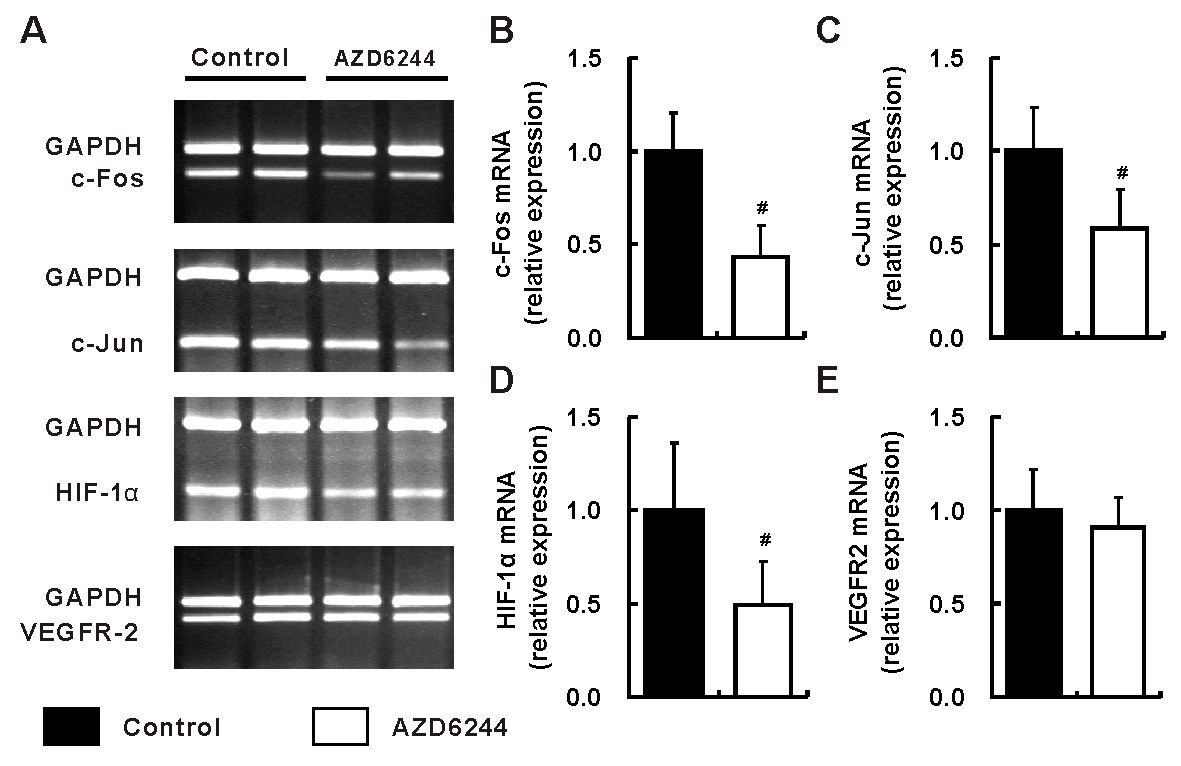


**Supporting figure 2 AZD6244 suppresses the integrated signal pathways in SGC7901 xenografts**

The mRNA level was measured by RT-PCR (A). Compared with control group, the mRNA of c-Fos (B), c-Jun (C) and HIF-1α (D) was substantially decreased in AZD6244 group, whereas, the mRNA of VEGFR-2 was comparable between two groups (E). #*p*<0.05 *vs*. control group.


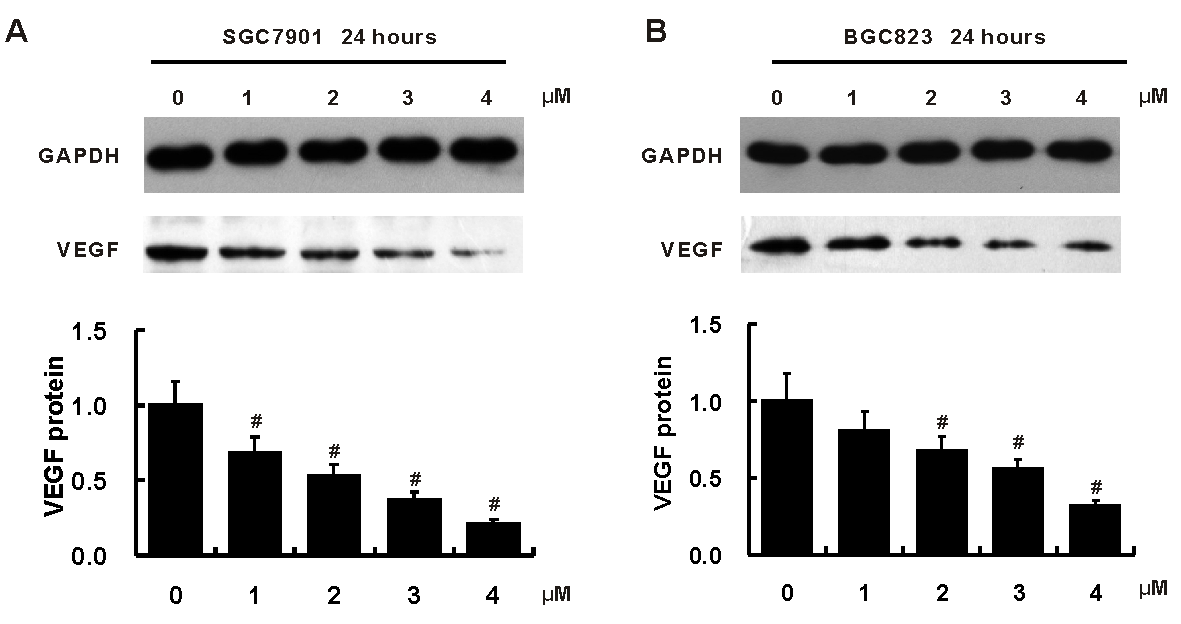


**Supporting figure 3 AZD6244 inhibits VEGF expression in SGC7901 and BGC823 gastric cancer cell lines**

Cells were treated with AZD6244 in the concentration of 0, 1, 2, 3, 4 μM for 24 hours, and then protein level of VEGF was determined by Western blot. AZD6244 at a concentration of 1, 2, 3, 4 μM was capable to suppress VEGF protein expression of SGC7901 cells (A). AZD6244 at a concentration of 2, 3, 4 μM but not 1 μM was able to inhibit VEGF protein expression of BGC823 cells (B). #*p*<0.05 *vs*. vehicle treated cells.
